# Case of Fungus of the Inferior Maxillary, Successfully Treated

**Published:** 1845-08

**Authors:** D. Harrington

**Affiliations:** Chestnut Street; Philadelphia, Dentist


### *Case of Fungus of the Inferior Maxillary, successfully Treated.*

By D. Harrington, of Philadelphia, Dentist.

The following case of maxillary disease, from its origin to its removal or permanent cure, was one of unusual occurrence, the circumstances connected with it being duly considered.

The young lady whose case is the subject of these remarks, Miss M. W., of the Northern Liberties, adjoining this city, was of good constitution, and had been free from any and every symptom of disease, prior to the appearance of this local affection. Her family, as far as known, have been uniformly in the enjoyment of excellent health ; of course she could not have been subject to any morbid inheritance.

The following is a concise description, from her own pen, of the rise and progress of the disease, and the remedial operations resorted to, together with their results up to the time of placing herself under my care.

"Some time during the month of April, 1842, I was much troubled with a very severe pain in my back teeth and jaw, on the left side below. I suffered much for some days, and then concluded to have the supposed cause of my affliction removed; and accordingly went to a Dentist and had two of my lower jaw teeth extracted. After the lapse of a few days there appeared upon the surface of the still lacerated gum, a small spongy tumour of a livid hue, which was immediately showed to the Dentist, who lanced it; after which it again increased in size, though no pain attended it. After a couple of days, such was its rapid growth, it was necessary to have it cut again—which was repeated once every two days for a couple of weeks. All this, however, appeared only to accelerate its growth; and becoming, in consequence, quite alarmed, I consulted our family physician, who pronounced it to be a fungus of a very alarming character, and that its proximity to certain parts rendered its extirpation by the knife, (the only alternative in his judgment,) a very critical, if not a very dangerous operation. This opinion, candidly expressed as it was, excited in my mind a great deal of alarm, indeed. Prompted by an excusable desire to obtain other advice, I called upon a Surgeon, of high standing in his profession, who requested that our family physician, above alluded to, should meet in consultation; the result of which consultation I did not learn. But he gave it as his opinion that, if it could be cured at all, it could be done without the knife—in other words, that it could not be cured by the knife. He then resorted to the application of a powerful caustic, for two or three weeks, but without any beneficial effect. Under the most fearful apprehensions of danger, caused by the surprising rapidity with which the fungus sprung up, (for it now nearly filled my mouth, and almost precluded the possibility of swallowing,) I resolved to yield to the application of the knife, as a dernier resort, and in accordance with the flattering advice of a second Surgeon of distinguished celebrity in his profession, but, I confess, with little faith on my own part in its utility. On the 25th of June, my family and our family physician concurring in opinion, the Surgeon last mentioned removed the fungus and a large portion of the *upper part* of the jaw bone on the left side, as far back as it was practicable. The pain of the operation, together with the great flow of blood, left me in a very weak state, and confined me to my bed for two weeks. After this I was able to open my mouth, so far only as to permit a partial examination of the state of my case; and, to the great surprise of all parties, the fungus was discovered to have sprung up again, nearly to its original size. This distressing circumstance seemed to prove conclusively to the mind of the Surgeon who had performed the operation, the absolute necessity of removing the whole of the jaw bone, from far forward, and as far back as the angle, if not to the condyle, on the left side. After some days I experienced a very severe pain in the right side of the under jaw, which, on being mentioned to the Surgeon, induced him to believe that the disease was of a more malignant and extensive nature than he had at first supposed: still the operation of removal of the left side of the lower jaw was not given up in idea.

"About this time the Rev. Dr. C., of whose congregation the most of my family were members, advised me to consult a Dentist with whom he had long been acquainted, and of whose skill he had a favourable opinion—and whose name I shall now freely mention, as I am through his aid, as an instrument in the hands of a merciful Providence, a radically cured woman. In accordance with the advice of Rev. Dr. C., I called on Mr. Harrington, Dentist. Mr. H. examined my case and pronounced it a very doubtful one, touching a cure. I told him of the wish of my last Surgeon to remove the major part of the jaw, &c. He advised me to submit to no such operation, unless I could be put into a sound magnetic sleep; he said the pain of such an operation would be excessive, without any tolerable certainty of a cure—but if I could be put into the sleep and have the operation performed without present pain, as he was sure many surgical operations had been done in various parts of Europe, and, in a few instances, on this side of the Atlantic,—the ridicule of *wise men*, and their denial notwithstanding—he would advise me to venture it, as a last resort, and at the same time be prepared for any consequences that might ensue; as the case, as related to a favourable result, was shrouded with doubt, in every view that could be taken of it. Mr. H. kindly offered me such facilities as were in his power, and I was tried, by the most powerful magnetisers that were to be found, between twenty to thirty times, to be placed in a full magnetic sleep, but without success. After this, some conversation about putting myself into his hands was had; but at this moment a medical gentleman of this city thought he could cure me, and kindly offered to make the trial, and Mr. H. strongly advised me to let him make the attempt. He commenced, and I remained under his treatment during five to six weeks, without success, for at the end of this time I was on the eve of suffocation, from the size and extension of the fungus into the roof of the mouth and down my throat. I now returned to Mr. Harrington, much worse than he had ever seen me before; who kindly received me into his family, that he might see me every hour, and have my case under his immediate observation and control.

M. W."

Near the end of the year 1842 Miss M. W. came under my care, and, as said by herself, her case had then become much worse than I had seen it at any former period; it being a truly malignant case of a mixed character, presenting the usual appearance of fungus hæmatodes in many places, and ordinary cancerous exterior in others. Her general system was also sympathising with her local affection in no small degree, in consequence of the vitiated blood of the fungus passing into the general circulation. Her face at this time presented the unhealthy hue of pale mahogany. Her buccinator and temporal muscles, parotid gland, &c., had become enormously enlarged, and withal so extremely rigid and unyielding, that her jaws could not be extended apart more than one-third of the usual distance when in health. The upper surface of the diseased jaw that had been left after the operation above mentioned by Miss W., had become distended, apparently, to three times its usual width, from near the symphysis back to its angle. The whole of this extensive surface sent up an extremely morbid and luxuriant growth, in which the teeth of the superior jaw became embedded and concealed, whenever the teeth on the opposite side were brought into contact. This morbid and fetid growth extended to the centre of the inferior jaw, and was strongly attached to the lining membrane of the mouth, pressing the tongue to the opposite side, and greatly impeding its proper and necessary offices. On the posterior part it was strongly attached to the constrictor muscles and side of the tongue, and extended far into the pharynx, nearly enclosing and concealing the uvula; and from Miss W.'s remark about being "on the eve of suffocation," was evidently pressing upon the epiglottis. Under circumstances of this distressing nature, there was no time to be lost in the application of such remedial means as the case might admit of. The immediate removal of the posterior portions of the fungus was attempted and performed, by drawing it upwards by the aid of a wire loop, and cutting it away in *small pieces*, so as to avoid hæmorrhage, and at the same time injury to important parts. This was, however, a very difficult operation to perform successfully, in consequence of the very small distance the teeth in front could be separated, and the enlargement of the posterior part of the tongue, above and beyond which the knife had to pass in operating. The excision in this case was performed by little and little, day after day, for several weeks in succession, for the purpose, as above said, of avoiding troublesome and dangerous hæmorrhage, as it would have been impossible to use an actual or potential cautery with success, if called for. I had another reason for this very gradual extirpation, by the knife, of the excrescence which, for several weeks, merely allowed of easy respiration, and a reception of food into the œsophagus. I could not, for a moment, suppose that local operations or applications (alone) could be made to accomplish much in the way of a radical cure, judging from the result of Miss W.'s past experience, and the contaminated state of her general system, until appropriate remedies, by way of the stomach, could be made to act *vigorously* and *simultaneously* with local ones. To accomplish this much desired, but almost hopeless result, viz.: a perfect cure of the case, the following mode of procedure was resorted to, and continued for several weeks:—Galvanic electricity, by placing the positive pole within the mouth upon the fungus, and making the negative pole to move constantly upon the exterior surface of the left side of the face, for and during ten to fifteen minutes each hour throughout the day, for two to three weeks. This was uniformly applied with that degree of intensity that the patient could conveniently sustain. Sarsaparilla, in a concentrated form, was given in large quantities and long continued—(iodine having been previously given while the patient was in other hands, without any evidence of beneficial effect.) The whole surface of the left side of the face was continually poulticed with wormwood, softened by simmering it in vinegar.

About one month from the commencement with these means, accompanied by slight cuttings from day to day, and the constant use of powerful astringents held in the mouth, there was an apparent improvement, both locally and generally, that seemed to point to a return of healthy action. This appearance was gratifying in a high degree, as it had the tendency to inspire a new degree of confidence in the appropriateness and efficacy of the means employed. It was now desirable to diminish the size of this fungus as fast as possible, for, notwithstanding the frequent cuttings, such had been its rapid growth after each operation, that it was nearly of the original dimensions; and what was truly alarming, its radicles had extended to, and attached themselves to new parts of the membrane of the mouth and tongue, and, posteriorly, near to the œsaphagus.

It will be seen from the above, that such was the inveterate disposition of this fungus to rapid increase, that it was necessary to accompany the knife with some application that would, at least, *resist* its luxuriant growth for the time being. This was a desideratum that involved much difficulty. Astringent washes had been tried and found useful, but not sufficiently so; the usual escharotics also had been made to accomplish very little, and for obvious reasons; these latter, in any of their known forms, could be kept in place but a few minutes, before the copious flow of saliva would, unavoidably, deprive their active principle of all energy, and render them inert. Various *new* articles were tried with little or no success. At length, *ashes* made from cast off stems of tobacco, put into thin linen sacks, so formed as to cover, as far as possible, the upper surface of the fungus, and be held in place from hour to hour by the pressure upon it of the teeth of the superior jaw, were found to operate like a charm, although not very charming in the production of pleasant feelings, for its caustic action was powerful, and would continue for several hours; the inflowing of the saliva notwithstanding. Two of these sacks were prepared for each night—one put in place on retiring, and replaced by the other about the middle of the night.

The possibility of a cure in Miss W.'s case now began to wear a more favourable aspect. With the exception of the galvanizing process, the other means that had been found appropriate and efficacious, viz.: the alterative given by the stomach, moderate cuttings daily, and the tobacco ashes occasionally, succeeded by an astringent and antiseptic wash of nutgalls, the wormwood poultice, and moderate attention to regimen, were carefully and systematically attended to for three to four months, during which the fungus contracted its circumference by very slow (almost imperceptible) degrees, and now covered principally the upper surface of the left side of the inferior maxilliary, (which, as before stated, was expanded to three times its proper width.) An opening was now discovered quite through the remaining portion of the jaw, near the angle; and a sinus extending from this opening, anteriorly, under the fungus, nearly to the symphysis. These openings were treated with astringent, and occasionally with acid injections, and sometimes filled with tobacco ashes.

Appearances were now so flattering that it was thought admissible for her to return to her home and friends, and by their aid continue the systematic application of the remedies ; calling once or twice a week to have her case examined. At the end of twelve months from the time Miss W. came under my care, her appearance, in all respects, afforded the most conclusive evidence of a return to perfect health, locally and generally; and, but for a remaining *enlargement* of the left side of her inferior maxillary, (and which, in all probability, will be years in disappearing, if it should during life,) it could not be discovered that she had ever been afflicted.

With reference to the case under consideration, it has been my lot to see, during my studentship with my highly respected friend and preceptor—the late Doctor H. H. Hayden, of Baltimore—and during an extensive practice in this city, many cases of the fungous and excrescent tribes, affecting the mouth, tongue, and their posterior collaterals : but I have never met with one that seemed, in the origin, to be so little under surgical and remedial control, and withal so malignant and extensive in its character. The too confident reliance on extirpation by the knife and powerful caustics, to the neglect of constitutional means, with the futile hope of accomplishing much in a very short time, it is probable, has been a common and fruitful cause of failure in the treatment of such affections.

With reference to my new escharotic, tobacco stem ashes, I am willing to believe it to be a valuable addition to the caustic class of remedies, especially in the management of excrescent affections of the mouth, vagina, rectum, &c., as it can be placed in a cavity and made to operate, for hours in succession, upon a morbid surface, without at the same time doing injury to healthy parts. To accomplish this, the sack for containing the ashes should be made of an impervious material in all of its parts, except where intended to cover and act upon the diseased surface.

In conclusion, I have entered more into detail in the above narrative than may, on a cursory view of the case, seem necessary, as I have never seen, or read of a case of a similar character, where the openings into the trachea and œsophagus were so obstructed by such a malignant mass of preternatural flesh, attached, withal, to so large a portion of the lining membrane of the pharynx. A large portion of the various kinds of fungi that have assailed the human mouth, far less in their dimensions and malignity than the above described, have terminated in a lingering and painful death.

Chestnut Street, Philadelphia.
*July* 23, 1845.

